# Melatonin supplementation does not alter vascular function or oxidative stress in healthy normotensive adults on a high sodium diet

**DOI:** 10.14814/phy2.15896

**Published:** 2023-12-18

**Authors:** Macarena Ramos Gonzalez, Michael R. Axler, Kathryn E. Kaseman, Andrea J. Lobene, William B. Farquhar, Melissa A. Witman, Danielle L. Kirkman, Shannon L. Lennon

**Affiliations:** ^1^ Department of Kinesiology and Applied Physiology University of Delaware Newark Delaware USA; ^2^ Department of Kinesiology and Health Sciences Virginia Commonwealth University Richmond Virginia USA

**Keywords:** brachial artery FMD, melatonin, NIRS, oxidative stress, sodium

## Abstract

High sodium diets (HSD) can cause vascular dysfunction, in part due to increases in reactive oxygen species (ROS). Melatonin reduces ROS in healthy and clinical populations and may improve vascular function. The purpose was to determine the effect of melatonin supplementation on vascular function and ROS during 10 days of a HSD. We hypothesized that melatonin supplementation during a HSD would improve vascular function and decrease ROS levels compared to HSD alone. Twenty‐seven participants (13 M/14 W, 26.7 ± 2.9 years, BMI: 23.6 ± 2.0 kg/m^2^, BP: 110 ± 9/67 ± 7 mmHg) were randomized to a 10‐day HSD (6900 mg sodium/d) supplemented with either 10 mg of melatonin (HSD + MEL) or a placebo (HSD + PL) daily. Brachial artery flow‐mediated dilation, a measure of macrovascular function, (HSD + PL: 7.1 ± 3.8%; HSD + MEL: 6.7 ± 3.4%; *p* = 0.59) and tissue oxygenation index (TSI) reperfusion rate, a measure of microvascular reactivity, (HSD + PL: 0.21 ± 0.06%/s; HSD + MEL: 0.21 ± 0.08%/s; *p* = 0.97) and TSI area under the curve (HSD + PL: 199899 ± 10,863 a.u.; HSD + MEL: 20315 ± 11,348 a.u.; *p* = 0.17) were similar at the end of each condition. Neither nitroxide molarity (HSD + PL: 7.8 × 10^−5^ ± 4.1 × 10^−5^ mol/L; HSD + MEL: 8.7 × 10^−5^ ± 5.1 × 10^−5^ mol/L; *p* = 0.55) nor free radical number (HSD + PL: 8.0 × 10^15^ ± 4.4 × 10^15^; HSD + MEL: 9.0 × 10^15^ ± 4.9 × 10^15^; *p* = 0.51) were different between conditions. Melatonin supplementation did not alter vascular function or ROS levels while on a HSD in this sample of young healthy normotensive adults.

## 1 INTRODUCTION

The average sodium intake in the United States is ~3500 mg/day while the Dietary Reference Intakes recommend ≤2300 mg/day (Stallings et al., 2019). Concern over the consumption of a high sodium diet (HSD) has been typically related to increases in blood pressure (BP) and subsequent hypertension (HTN); however, multiple organ systems, such as the vasculature, are negatively affected as well (Elliott et al., 1989; Robinson et al., 2019). Indeed, our group demonstrated that a HSD impairs macrovascular and microvascular function independent of changes in BP (DuPont et al., 2013; Greaney et al., 2012). Increases in reactive oxygen species (ROS), in particular superoxide (O_2_^−^), have been shown to be one mechanism responsible for this impairment (Lenda et al., 2000; Lenda & Boegehold, 2002a, 2002b; Zhu et al., 2004). We previously demonstrated that ROS scavengers restored microvascular function in normotensive adults that underwent a 7‐day HSD (Greaney et al., 2012; Ramick et al., 2019). Furthermore, ROS have been linked directly to vascular disease as they decrease nitric oxide (NO) bioavailability (Munzel et al., 2017). A loss of NO can increase the risk of atherosclerosis (Forstermann & Munzel, 2006) and HTN (Hermann et al., 2006).

Melatonin is a hormone synthesized in the pineal gland predominantly at night and has been shown to have antioxidant properties (Tengattini et al., 2008). Numerous studies show that at least 1 month of melatonin supplementation reduces ROS in individuals with HTN, metabolic syndrome, or diabetes (Kedziora‐Kornatowska et al., 2007, 2008, 2009; Kozirog et al., 2011; Raygan et al., 2019). These antioxidant benefits are accompanied by improvements in endothelial function (Hung et al., 2013; Pogan et al., 2002; Salmanoglu et al., 2016; Shao et al., 2017). In vitro, melatonin has been shown to increase NO levels and expression of endothelial nitric oxide synthase (eNOS) in human umbilical vein endothelial cells cultured under pressure to simulate HTN (Shao et al., 2017). In vivo, melatonin has increased eNOS protein expression and prevented endothelial dysfunction in Sprague–Dawley rats under chronic hypoxia (Hung et al., 2013). Interestingly, a link exists between CVD and melatonin as evidenced by reductions in nocturnal synthesis, circulating levels of melatonin, and its urinary metabolite (6‐sultatoxymelatonin) in various CVD populations (Altun et al., 2002; Dominguez‐Rodriguez et al., 2014; Girotti et al., 2003). Furthermore, the incidence of cardiac events is higher in the early morning when circulating melatonin levels are lower (Bilo et al., 2018; Muller et al., 1987; Siegel et al., 1992). To date, there are limited studies on the effect of melatonin on the vasculature in humans, and no studies in adults consuming a HSD. It is also unknown whether melatonin administration can reduce ROS in humans consuming a HSD.

Therefore, the primary aim of this study was to determine the effect of melatonin supplementation during 10 days of a HSD on macrovascular and microvascular function. Our secondary aim was to determine the effect of melatonin during 10 days of a HSD on ROS levels. We hypothesized that melatonin supplementation during 10 days of a HSD would lead to greater vascular function and lower ROS compared to a HSD plus placebo.

## 2 MATERIALS AND METHODS

### 2.1 Participants

The study protocol was approved by the Institutional Review Board of the University of Delaware and conformed to the provisions of the Declaration of Helsinki. The study was registered on clinicaltrials.gov (NCT04325191). Written informed consent was obtained from all participants prior to participation. We recruited healthy men and women aged 18–45 years. Inclusion criteria included a BP <130/80 mmHg and body mass index (BMI) between 18.5 and less than 30 kg/m^2^. Participants were excluded for a history of CVD, liver or kidney disease, malignant cancer, sleep disorders, mood disorders, and/or taking any medications for those conditions. Those performing night shift work, use of tobacco products, ADHD medication use, melatonin, or other antioxidant use in the past 3 months, significant weight changes (≥10 lbs. gain or loss) in the last 6 months, women who were pregnant or breastfeeding, and highly trained endurance athletes were also excluded.

Participants completed a medical history questionnaire, a menstrual cycle form (women only), and a global physical activity questionnaire (GPAQ) (Armstrong & Bull, 2006) during the screening. Anthropometrics and seated BP (dominant arm; Dash 2000, GE medical systems, Chicago, IL) were measured. A venous blood sample was analyzed for a metabolic panel and lipid profile at the screening. Before beginning the study, participants recorded their habitual dietary intake for two weekdays and one weekend day. They were provided with a handout of two‐dimensional food models to help with estimating portion sizes. Diet records were reviewed by a trained researcher and analyzed using Nutrition Data System for Research software (NDSR, 2020, University of Minnesota, Minneapolis). These records were used to help counsel subjects to consume 2300 mg sodium per day during the study conditions.

### 2.2 Experimental protocol

This was a randomized double‐blind placebo‐controlled crossover study which consisted of two 10‐day conditions separated by a washout period of at least 14 days. During both conditions, participants consumed salt pills and supplemented with either 10 mg of melatonin (HSD + MEL) or a lactose placebo (HSD + PL) capsule daily. Melatonin and placebo capsules were manufactured by SaveWay compounding pharmacy (Newark, DE) and were matched in weight and appearance. The melatonin was purchased from Letco by Fargon Medical and had the USP designation. Participants were instructed to take the capsule 30 min before going to sleep. The dose of melatonin used has been shown to decrease ROS in clinical populations with no reported side effects (Raygan et al., 2019; Szewczyk‐Golec et al., 2017). The total intake of 6900 mg/day of sodium in both 10‐day interventions was achieved by consuming 2300 mg of sodium in the diet and by taking 12 enteric coated, slow‐release salt pills per day, each containing ~380 mg of sodium (4600 mg total). This level of sodium intake is consistent with our prior studies and shown to impair vascular function in normotensive adults (DuPont et al., 2013; Greaney et al., 2012). Participants were provided with instructions and strategies to consume a 2300 mg sodium diet during both conditions. Participants recorded their dietary intake during the last 3 days of the intervention (days 7–9) and were instructed to replicate their diet during both conditions.

#### 2.2.1 Sleep assessment

Participants wore wrist accelerometers (Motionlogger Micro watch, Ambulatory Monitory Inc, Tokyo, Japan) on their nondominant wrist for 9 days and nights, except for activities involving water. Data were collected in the zero‐crossing mode (ZCM) and saved in 1‐min epochs. The University of California San Diego algorithm, which is validated to produce accurate and reliable sleep estimates relative to polysomnography (Jean‐Louis et al., 2001), along with Action W‐2 software (Ambulatory Monitoring, Inc., Ardsley, NY) were used to score the data. Data were acceptable if the accelerometer was worn for at least 7 out of the 9 days (Ancoli‐Israel et al., 2015). Participants also completed the Pittsburgh Sleep Quality Index (PSQI), on the last day of each intervention.

#### 2.2.2 Urine collection

Participants collected their urine for 24 h starting on Day 9. Urine was assessed for volume, urine specific gravity (Goldberg Brix Refractometer, Reichert Technologies, Depew, NY), electrolyte concentrations (EasyElectrolyte Analyzer, Medica, Bedford, MA), and osmolality (Advanced 3D3 Osmometer, Advanced Instruments, ON, Canada). Urine flow rate was calculated and used to determine 24‐h sodium excretion. Urine collections were considered valid if collected within 20–28 h, there were not ≥2 missed collections, and volume was ≥500 mL. The first morning void was collected separately on Day 10 to assess melatonin compliance by measuring urinary 6‐sulfatoxymelatonin (ELISA kit, Alpco, Salem, NH). A 4 Parameter Logistics curve fit was used to obtain the calibrator curve with SoftMax Pro Software (Molecular Devices). Values at or above the maximum detectable level of the assay were assigned that value for statistical analysis purposes.

### 2.3 Experimental visit

Experimental visits were scheduled in the morning (7 am–10 am) of Day 10 and at the same time for both conditions. Participants were asked to fast for at least 9 h, avoid caffeine and alcohol for at least 12 h, and not exercise for 24 h. Upon arrival, body mass was measured. After 5 min of rest, seated BP was assessed in triplicate and averaged (dominant arm; Dash 2000, GE medical systems, Chicago, IL). A venous blood sample was collected to assess hematocrit (Thermo Sorvall), hemoglobin (Hb 201+, HemoCue, Angelholm, Sweden), serum electrolytes (EasyElectrolyte Analyzer, Medica, Bedford, MA), plasma osmolality (Advanced 3D3 Osmometer, Advanced Instruments, ON, Canada), and the lipid profile (LabCorp).

#### 2.3.1 Macrovascular function

Brachial artery flow‐mediated dilation (FMD) was performed according to established guidelines as an assessment of macrovascular function (Thijssen et al., 2019). After the participant rested for 20 min in a supine position, longitudinal images of the brachial artery and Doppler blood velocities were recorded proximal to the antecubital fossa using a 10 MHz linear phased array ultrasound transducer (Logiq e, GE, Boston, MA). After recording baseline images for 1 min, a BP cuff placed just below the antecubital space was rapidly inflated to 200 mmHg for 5 min (AG101 rapid cuff inflator, Hokanson, Bellevue, WA). After cuff release, images and blood velocity were recorded for 2 min during this period of reactive hyperemia. Images were analyzed across complete heart cycles using automated edge‐detection software (Cardiovascular Suite, Quipu, Italy). Baseline diameter was determined as the average diameter recorded over the baseline period. Peak diameter was determined as the greatest 3‐sec rolling average diameter achieved during reactive hyperemia. Absolute FMD was determined as the difference between peak and baseline diameter. Relative FMD was determined as the absolute FMD expressed as a percentage of the baseline diameter. Shear rate area under the curve (AUC) from cuff release to peak diameter was calculated.

#### 2.3.2 Microvascular function

Near‐infrared spectroscopy (NIRS) and a vascular occlusion test (VOT) were performed as a test of microvascular function following established guidelines (Barstow, 2019). The NIRS device (PortaMon; Artinis, Elst, The Netherlands) was placed on the proximal/medial portion of the forearm and secured with a black neoprene sleeve to prevent movement and external light interference. Oxysoft software (Artinis) was used for data acquisition (10 Hz). After recording baseline values for 3 min, a BP cuff placed just above the elbow was rapidly inflated to 200 mmHg for 5 min (AG101 rapid cuff inflator, Hokanson, Bellevue, WA). Data were recorded for 5 min following cuff release. The NIRS‐VOT was used to assess local tissue saturation expressed as the tissue saturation index (TSI). Slope 1 or the desaturation slope represents the reduction in regional tissue oxygenation during cuff inflation. Slope 2 or the resaturation slope represents the postischemic hyperemia phase in which regional tissue oxygenation increases during cuff deflation. Baseline TSI was calculated as the average TSI 1 min before the onset of arterial cuff occlusion. Microvascular reactivity was determined as TSI resaturation slope and quantified with linear regression as the average upslope from cuff deflation until the participant reached their respective baseline value. The reperfusion area under the curve (TSI AUC) was determined from the baseline value achieved during reactive hyperemia until the end of the test.

#### 2.3.3 Oxidative stress measurement

Electron paramagnetic resonance (EPR) was used to measure O_2_^−^ in whole blood samples collected on Day 10 using the EMXnano (Bruker). Blood was processed immediately after it was drawn at the beginning of the experimental visit. Whole blood samples were incubated in a buffer solution containing phosphate buffered saline, diethylenetriaminepentaacetic acid (DTPA) (100 μM), and 1‐hydroxy‐3‐methoxycarbonyl‐2,2,5,5‐tetramethyl‐pyrrolidine (CMH) (0.2 mM) for 10 min at 37°C. CMH reacts with O_2_^−^ to form nitroxide CM, a stable free radical detected by EPR. Following incubation, samples were flash‐frozen in liquid nitrogen and stored at −80°C. The EPR acquisition parameters include microwave frequency = 9.65 GHz; center field = 3436 G; sweep width = 150 G; sweep time = 20 s; microwave power = 0.316 mW; receiver gain = 40 dB; modulation amplitude = 3.0 G; number of scans = 4; microwave attenuation = 35 dB; and time constant = 10.24 ms (Elajaili et al., 2019). All samples were measured in triplicate and averaged. O_2_^−^ concentration was quantified as nitroxide molarity and number of free radicals which is directly proportional to the amount of ROS in the sample.

### 2.4 Statistical analysis

Our primary outcome was brachial artery FMD measured on Day 10 of each condition. Secondary outcomes included TSI reperfusion slope and TSI AUC acquired from the NIRS‐VOT. An a priori power analysis determined that a sample size of 21 participants provided 95% power to detect a difference in FMD of at least 1% (effect size 0.83) at the end of the two arms using a paired samples *t*‐test with an alpha of 0.05 (G* Power). This difference in FMD was based on our results demonstrating that dietary potassium attenuated the effect of sodium (Smiljanec et al., 2020). An improvement of 1% in FMD is clinically significant based on a recent meta‐analysis that found a 1% improvement in FMD was associated with a decrease of 8%–10% in overall CVD risk over 4 years (Ras et al., 2013). Comparisons between the two conditions for all variables were assessed using paired *t*‐tests. Although not powered to detect sex differences, an exploratory analysis was conducted to examine potential differences between men and women in their response to the two conditions. Therefore, the difference score was calculated for the primary outcomes for men and women and was compared using an unpaired t‐test. Data were assessed for normality prior to analysis. JMP Pro 16.0.0 (SAS, Cary, NC) was used to carry out the analysis. Significance was set at *p* < 0.05.

## 3 RESULTS

Participant screening characteristics and blood chemistries for the 27 participants who completed this study are presented in Table 1. We had a near equal distribution of men and women who were mostly Caucasian and Asian. Overall, participants were young and normotensive with a nonobese BMI. Screening blood work was within normal limits. Men were heavier, taller, and had a higher screening systolic BP. Habitual intake of our participants indicated that they consumed approximately 1.6 times the Dietary Reference Intake for sodium (2300 mg) which is consistent with U.S. average intake for adults (see Table S1). Results derived from the GPAQ indicated that participants were moderately active (average of 5.3 ± 2.8 h of physical activity per week), while they spent 8.5 ± 3.3 h per day sitting or reclining.

**TABLE 1:** Screening characteristics.

	All subjects	Men	Women	*p* Value
N	27	13	14	
Age, year	26.7 ± 2.9	27.2 ± 2.0	26.3 ± 3.6	0.45
Race, Asian/Black/Caucasian	13/1/13	7/1/5	6/0/8	
Body Mass, kg	68.2 ± 11.1	76.1 ± 10.8	60.9 ± 4.4	<0.0001
Body Height, cm	169.4 ± 9.9	177.0 ± 7.33	162.4 ± 6.2	<0.0001
BMI, kg/cm^2^	23.6 ± 2.0	24.2 ± 2.1	23.1 ± 1.9	0.19
Systolic BP, mm Hg	110 ± 9	115 ± 9	105 ± 6	0.001
Diastolic BP, mm Hg	67 ± 7	69 ± 7	64 ± 5	0.06
Heart rate, bpm	68 ± 12	66 ± 12	71 ± 13	0.30
Hemoglobin, g/dL	13.9 ± 1.4	15.1 ± 0.8	12.8 ± 1.0	<0.0001
Hematocrit, %	41.8 ± 4.4	45.5 ± 2.4	38.3 ± 2.6	<0.0001
Glucose, mg/dL^a^	75.3 ± 16.1	74.2 ± 17	76.2 ± 16.0	0.76
BUN, mg/dL	12.2 ± 3.6	14.1 ± 4.1	10.5 ± 2.0	0.01
Creatinine, mg/dL	0.8 ± 0.2	0.97 ± 0.08	0.72 ± 0.13	<0.0001
eGFR, mL/min/1.73	110.0 ± 13.7	107.8 ± 10.8	112.0 ± 16.0	0.64
Sodium, mmol/L	140.8 ± 1.7	141.1 ± 1.6	140.5 ± 1.9	0.44
Chloride, mmol/L	102.4 ± 2.3	101.6 ± 2.3	103.1 ± 2.2	0.11
Total Cholesterol, mg/dL	187.2 ± 29.2	182.8 ± 28.3	191.2 ± 30.5	0.48
LDL Cholesterol, mg/dL	109.8 ± 28.1	111.8 ± 23.6	107.9 ± 32.6	0.73
VLDL Cholesterol, mg/dL	17.2 ± 6.8	19.6 ± 7.7	15.0 ± 5.1	0.09
HDL Cholesterol, mg/dL	60.2 ± 14.2	51.3 ± 10.6	68.4 ± 12.2	0.001
Triglycerides, mg/dL	94.0 ± 39.6	106.5 ± 44.2	82.5 ± 32.3	0.13

Abbreviations: BMI, body mass index; BP, blood pressure; BUN, blood urea nitrogen; eGFR, estimated glomerular filtration rate; HDL, high‐density lipoprotein; LDL, low‐density lipoprotein; MAP, mean arterial pressure; VLDL, very‐low‐density lipoprotein.

*Note*: Data are expressed as means ± SD. *N* = 27. *p* values compare men and women.

^a^ *n* = 24.

Table 2 presents anthropometrics, hemodynamics, blood, and urine results from the experimental visits on Day 10. There were no differences between conditions for any of these variables. Twenty‐four‐h urinary sodium excretion was elevated and similar in both conditions. Urinary 6‐sulfatoxymelatonin was greater during the melatonin condition demonstrating that the supplementation was successful in increasing melatonin levels. Participants reported their dietary intake data on days 7–9 of each condition. Participants consumed similar amounts of energy (HSD + PL: 2037 ± 715 kcal/d; HSD + MEL: 1915 ± 530 kcal/d; *p* = 0.31) and sodium (HSD + PL: 3346 ± 1456 kcal/d; HSD + MEL: 3190 ± 1298 kcal/d; *p* = 0.62) demonstrating that they followed a similar diet during both conditions (see Table S2).

**TABLE 2:** Participant characteristics on Day 10 of each intervention.

	HSD + PL	HSD + MEL	*p* Value
Body Mass, kg	70 ± 11	69 ± 11	0.38
Systolic BP, mm Hg	113 ± 9	114 ± 8	0.82
Diastolic BP, mm Hg	69 ± 7	69 ± 7	0.62
MAP, mm Hg	84 ± 7	84 ± 7	0.77
Heart rate, bpm	73 ± 12	72 ± 13	0.47
Serum Sodium, mmol/L^a^	139.9 ± 1.9	140.3 ± 2.9	0.79
Serum Chloride, mmol/L^a^	106.0 ± 1.6	106.2 ± 2.6	0.92
Plasma osmolality, mOsm/kg/H_2_O^b^	291.2 ± 7.6	289.9 ± 4.9	0.53
Hemoglobin, g/dL^a^	13.7 ± 1.7	13.5 ± 1.4	0.44
Hematocrit, %^a^	41.7 ± 4.5	41.1 ± 3.9	0.41
Total Cholesterol, mg/dL^b^	174.2 ± 31.6	173.7 ± 35.3	0.89
LDL Cholesterol, mg/dL^b^	100.2 ± 24.4	99.3 ± 30.5	0.73
VLDL Cholesterol, mg/dL^b^	17.5 ± 7.4	16.8 ± 6.7	0.40
HDL Cholesterol, mg/dL^b^	56.5 ± 14.2	57.6 ± 16.1	0.32
Triglycerides, mg/dL^b^	94.6 ± 46.4	90.1 ± 39.6	0.41
Urinary sodium excretion, mmol/24h^c^	317.9 ± 97.3	299.9 ± 97.3	0.59
Urine osmolality, mOsm/kg^c^	556.5 ± 208.6	530.6 ± 234.4	0.46
6‐sulfatoxymelatonin, ng/mL^a^	48.5 ± 31.9	373.2 ± 90.7	0.001

Abbreviations: BP, blood pressure; HDL, high‐density lipoprotein; HSD + MEL, high sodium diet plus melatonin; HSD + PL, high sodium plus placebo (lactose); LDL, low‐density lipoprotein; MAP, mean arterial pressure; VLDL, very‐low‐density lipoprotein.

*Note*: Data are expressed as means ± SD and were analyzed using paired *t*‐tests; *n* = 27.

^a^ *n* = 26.

^b^ *n* = 23.

^c^ *n* = 25.

**FIGURE 1:**
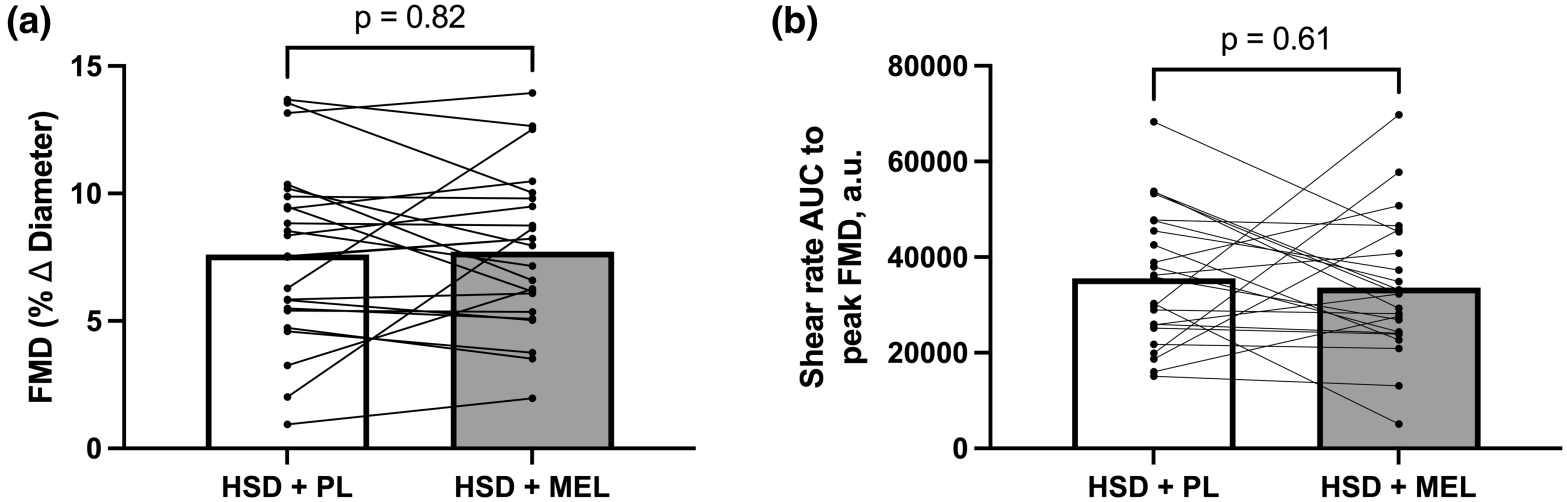
Macrovascular function assessed via brachial artery flow‐mediated dilation (FMD) at the end of the high sodium diet plus placebo condition (HSD + PL) and at the end of the high sodium diet plus melatonin condition (HSD + MEL). (a) Relative FMD; (b) Shear rate area under the curve (AUC) to peak reached during reactive hyperemia. *n* = 22. Data were analyzed using paired *t*‐tests and are presented as both the average and individual data points.

**FIGURE 2:**
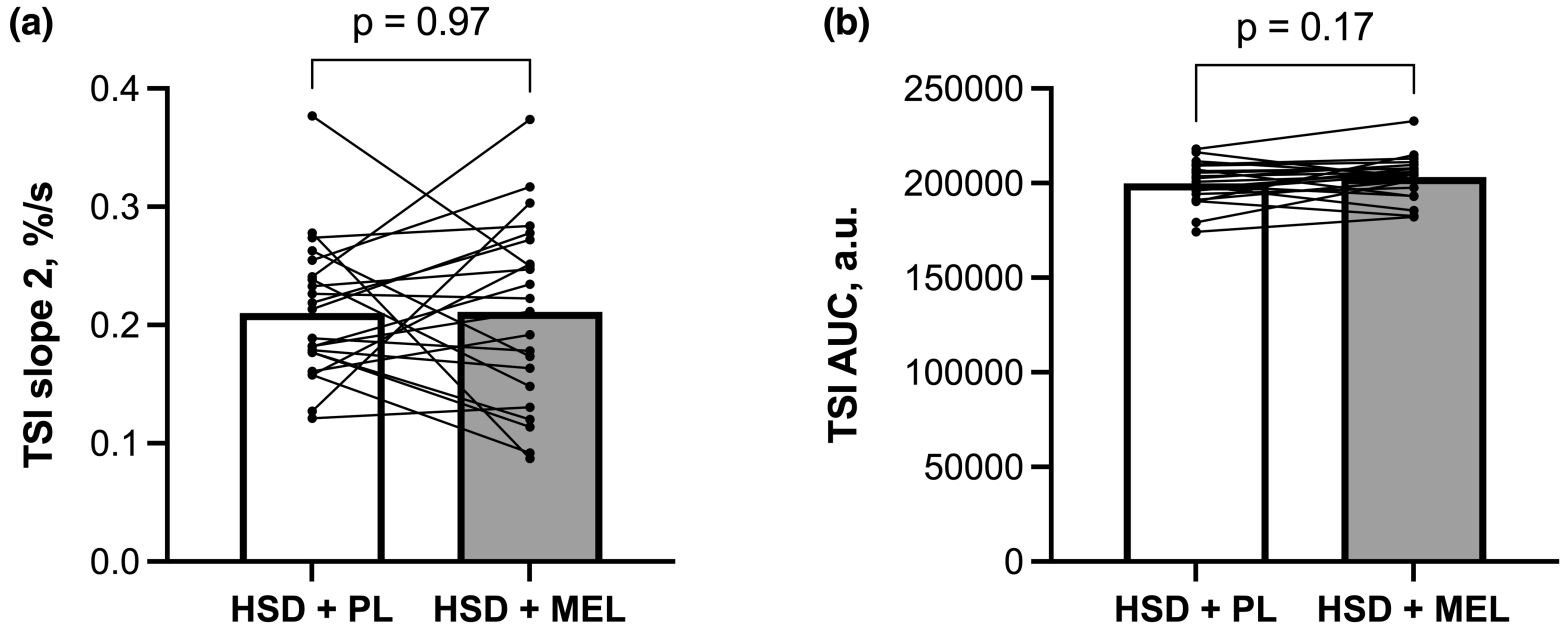
Microvascular function assessed via near‐infrared spectroscopy during vascular occlusion test (NIRS‐VOT) at the end of the high sodium diet plus placebo condition (HSD + PL) and at the end of the high sodium diet plus melatonin condition (HSD + MEL). (a), Slope 2, also known as reperfusion slope; (b) Tissue saturation index area under the curve (TSI AUC). *n* = 22. Data were analyzed using paired *t*‐tests and are presented as both the average and individual data points.

Vascular function results collected on Day 10 of each condition are presented in Table 3, Figure 1, and Figure 2. Macrovascular function was assessed using brachial artery FMD. We did not observe any differences in baseline brachial artery diameter at the end of each condition (Table 3). Furthermore, we did not observe any differences in FMD or shear rate AUC (Figure 1a,b). We analyzed the difference score between men and women for FMD and there was no difference (*p* > 0.05; data not shown). Microvascular function was assessed by NIRS‐VOT. There were no differences between baseline TSI nor were there any differences for either slope 2, the reperfusion slope or TSI AUC (Figure 2a,b) at the end of each condition. Nor was there a change in difference score between men and women for these variables.

**TABLE 3:** Vascular function on Day 10 of each condition.

	HSD + PL	HSD + MEL	*p* Value
Brachial artery FMD			
Baseline brachial artery diameter, mm	3.35 ± 0.66	3.37 ± 0.63	0.68
Peak brachial artery diameter, mm	3.60 ± 0.65	3.62 ± 0.64	0.58
Absolute FMD, mm	0.24 ± 0.10	0.25 ± 0.09	0.67
Time to peak diameter, s	45.5 ± 10.7	48.1 ± 13.1	0.41
NIRS‐VOT^a^			
Baseline TSI, %	65.6 ± 3.8	65.3 ± 3.9	0.72
TSI_min_, %	33.1 ± 6.8	34.6 ± 10.9	0.39
TSI_max_, %	75.9 ± 3.6	76.5 ± 3.0	0.41
Slope 1, %/s	−0.012 ± 0.003	−0.012 ± 0.004	0.66
Hyperaemic reserve, %	15.9 ± 5.1	17.3 ± 5.8	0.30

Abbreviations: AUC, area under the curve; FMD, flow‐mediated dilation; HSD + MEL, high sodium diet plus melatonin; HSD + PL, high sodium diet plus placebo (lactose); NIRS‐VOT, near‐infrared spectroscopy with vascular occlusion test; TSI, tissue oxygenation index; TSI_max_, maximum TSI; TSI_min_, minimum TSI.

*Note*: Data are expressed as means ± SD and were analyzed using paired *t*‐tests. *n* = 23.

^a^ *n* = 22.

**FIGURE 3:**
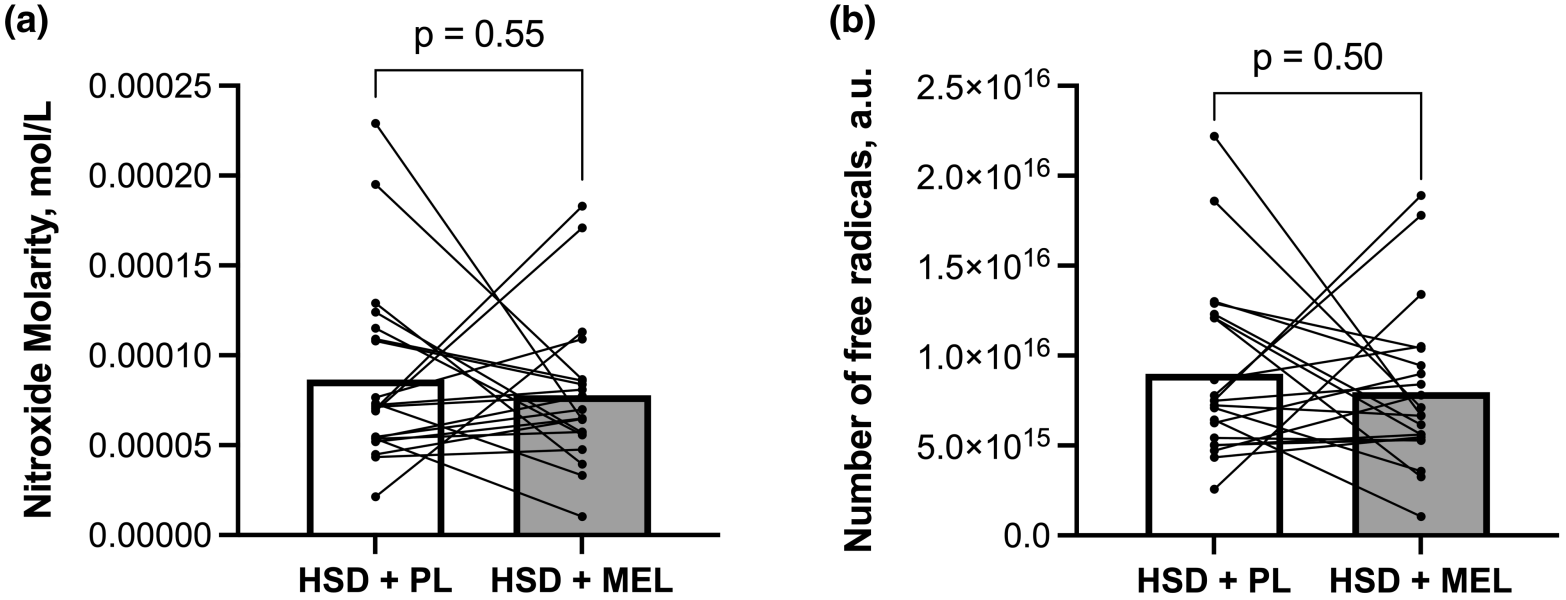
Reactive oxygen species (ROS) at the end of the high sodium diet plus placebo condition (HSD + PL) and the high sodium diet plus melatonin condition (HSD + MEL). (a) Nitroxide concentration; (b) total number of free radicals. *n* = 21. Data were analyzed using paired *t*‐tests and are presented as both the average and individual data points.

ROS were measured as a potential mechanism by which melatonin may exert its antioxidant effects. We found no difference in superoxide between conditions as assessed by nitroxide molarity and free radical number as depicted in Figure 3a,b. Nor we did find any significant changes in the difference score between men and women.

## 4 DISCUSSION

We performed a randomized controlled crossover trial in young, healthy, normotensive adults to evaluate the effect of melatonin during a HSD on vascular function. Melatonin has been shown to have antioxidant‐like properties and has been effective in reducing oxidative stress in clinical populations as well as improving cardiovascular biomarkers (Kedziora‐Kornatowska et al., 2007, 2008, 2009; Kozirog et al., 2011; Raygan et al., 2019). However, we found that daily melatonin supplementation for 10 days did not alter macrovascular or microvascular function in healthy adults consuming a HSD. Furthermore, there was no difference in superoxide levels between our melatonin and placebo conditions. Thus, contrary to our hypothesis, we did not find that melatonin supplementation altered vascular function or ROS levels in young adults consuming a HSD.

We measured vascular function following 10 days of a HSD, achieved using salt pills. Previous work using salt pills to increase sodium intake over 10 days has shown a reduction in brachial artery FMD in young healthy adults compared to a recommended sodium diet (Babcock et al., 2020). In our design, we did not have a low sodium control and therefore cannot evaluate whether FMD fell in response to the salt pills. However, we found that urinary sodium excretion values were consistent with expected sodium intake and with previous reports suggesting our participants were compliant with taking the salt pills (Babcock et al., 2020). Indeed, the focus of our study was to evaluate whether melatonin supplementation could increase FMD on a HSD diet compared to a HSD diet alone, which we did not observe. By contrast, animal and cell work has shown that melatonin improves vascular function in vivo and in vitro (Hung et al., 2013; Pogan et al., 2002; Salmanoglu et al., 2016; Shao et al., 2017). Melatonin restored endothelial function in diabetic rats (Salmanoglu et al., 2016) and rats with chronic intermittent hypoxia (Hung et al., 2013). It has also prevented renal injury in hypertensive rats fed a HSD (Leibowitz et al., 2016). Furthermore, in patients undergoing coronary artery bypass graft surgery, a month of melatonin supplementation decreased markers of endothelial damage (Javanmard et al., 2016) highlighting its benefit in a clinical population. By contrast, we studied healthy individuals who may be less responsive to melatonin's beneficial properties. Melatonin supplementation has been shown to have beneficial effects on blood pressure, platelet reactivity, vasomotor properties, and inflammation (Jiki et al., 2018) in middle age to older adults with clinical conditions.

Microvascular function was evaluated using NIRS‐VOT in addition to FMD. Changes in the microvasculature can often present before changes in larger conduit vessels highlighting the importance of examining the microvasculature (Holowatz et al., 2008). We have previously demonstrated a reduction in microvascular function after 7 days of a HSD using cutaneous local heating in healthy young adults (Greaney et al., 2012; Ramick et al., 2019). We did not find any differences in microvascular function using NIRS between the melatonin and placebo conditions. It is possible that our null vascular findings could be attributed to the timing of our experimental visits which occurred in the morning, when melatonin levels are lower (Bilo et al., 2018; Muller et al., 1987; Siegel et al., 1992). The average half‐life of melatonin has been reported to be 54 min with plasma melatonin levels returning to baseline approximately 5 h after consumption in young healthy males (Andersen et al., 2016). We did observe a morning increase in melatonin's urinary metabolite, which corresponds to a greater nocturnal plasma peak in the melatonin condition demonstrating that our participants took their melatonin capsules daily at night; however, plasma levels may have fallen by the time of the experimental visit.

We have previously demonstrated that ROS scavengers can mitigate impaired microvascular function imposed by a HSD using a controlled feeding study approach (Greaney et al., 2012; Ramick et al., 2019). Furthermore, Baric et al. (2020) demonstrated that oral supplementation of an antioxidant cocktail (vitamins C and E) prevented microvascular dysfunction induced by a 7‐day HSD and lowered markers of oxidative stress in young healthy individuals, although they did not utilize a randomized design. However, despite studies demonstrating antioxidant properties for melatonin (Acuna‐Castroviejo et al., 2014; Anwar et al., 2015; Pechanova et al., 2014; Tengattini et al., 2008), we did not observe any difference in ROS levels between the melatonin and placebo conditions. Melatonin has been shown to increase antioxidant enzymes and decrease ROS as seen by increases in SOD and catalase activity, and decreases in erythrocyte malondialdehyde levels in treated older adults with HTN and elderly adults with type II diabetes (Kedziora‐Kornatowska et al., 2008, 2009). Several potential reasons could explain the lack of difference in the ROS levels between conditions. Firstly, while our study used a higher dosage than other studies performed in clinical populations (10 mg compared to 5 mg), the latter studies lasted longer (1–2 months compared to 10 days) (Kedziora‐Kornatowska et al., 2008, 2009; Kozirog et al., 2011; Raygan et al., 2019). Secondly, most human studies have been performed in clinical populations. To date, there is limited evidence on the effect of melatonin on ROS in healthy individuals. One study in healthy males that underwent a strenuous 50 km endurance race showed a decrease in oxidative damage in those that had taken melatonin prior to the race (Ochoa et al., 2011). Thirdly, the methodology used to quantify ROS varies greatly and therefore comparisons between studies are difficult. We utilized EPR, which is considered a gold standard in assessment of ROS (Abdel‐Rahman et al., 2016).

### 4.1 Limitations

While we utilized a randomized, crossover design, we did not collect baseline measures and can only compare our outcomes on day 10. Due to this design, we did not control for the menstrual cycle phase in our female participants. Studies have shown no difference in FMD between distinct phases of the menstrual cycle in premenopausal women (Shenouda et al., 2018). Second, melatonin has been shown to increase the activity and concentration of endogenous antioxidant enzymes while sodium has been shown to decrease these. However, in this study we did not evaluate the effect of melatonin on endogenous antioxidants during the HSD. Furthermore, as our purpose was to compare melatonin supplementation to a placebo while consuming a HSD, we do not know whether the HSD alone caused vascular impairment and increased ROS as since we did not include a low sodium condition. Nonetheless, previous work using salt pills to increase sodium intake over 10 days has shown a reduction in brachial artery FMD in young healthy adults compared to a recommended sodium diet (Babcock et al., 2020). Third, while we could not quantify the change in plasma melatonin levels at night, we did assess melatonin's urinary metabolite in the morning, which is highly correlated with the former (Benloucif et al., 2008; Graham et al., 1998). Fourth, CMH has the greatest interaction with O_2_^−^, however, there is evidence it reacts with other radicals (Berg et al., 2014; Dikalov et al., 2007). Therefore, we report concentrations of nitroxide. Lastly, our sample consisted of young healthy normotensive adults who may buffer sodium‐induced oxidative stress more effectively and therefore, do not need an endogenous source of antioxidants. Therefore, the results should not be extrapolated to other populations.

## 5 CONCLUSIONS

In conclusion, our findings showed that 10 days of melatonin supplementation (10 mg/d) did not improve macrovascular or microvascular function compared to a placebo and did not decrease ROS in our sample of young healthy normotensive adults consuming a HSD. Future studies should investigate different melatonin doses and durations, explore mechanisms beyond ROS formation, such as inflammation and antioxidant defense systems, and examine other populations.

## AUTHOR CONTRIBUTIONS

M.R.G., S.L.L., W.B.F, M.A.W., and D.L.K. conceptualized and designed the experiments. M.R.G., M.R.A., K.E.K., and A.J.L. performed the experiments. M.R.G and S.L.L. analyzed and interpreted the data. M.R.G. drafted the original manuscript. M.R.G., S.L.L., and A.J.L. contributed to the statistical analysis. All authors reviewed and approved the manuscript.

## FUNDING INFORMATION

This research was supported, in part, by a National Institutes of Health (NIH) grant HL145055 and P20 GM113125.

## ETHICS STATEMENT

Each participant provided written informed consent as approved by the University of Delaware Institutional Review Board.

## Supporting information


Table S1

Table S2

## Data Availability

Data are available upon reasonable request to the principal investigator after institutional data transfer agreement approvals.
