# Supplementary material for: Melatonin supplementation does not alter vascular function or oxidative stress in healthy normotensive adults on a high sodium diet
Source: Physiol Rep. 2023 Dec 18;11(24):e15896. doi: 10.14814/phy2.15896 (PMC10727961; doi:10.14814/phy2.15896)
Supplement: Supplementary file 1 — Table S1 [file PHY2-11-e15896-s001.docx]

**Supplemental Table 1**. Participants Habitual Dietary Intake

|  |  |
| --- | --- |
| Energy intake, kcal/day | 2087 ± 512 |
| Total carbohydrate, g/day | 246 ± 74 |
| Total protein, g/day | 96 ± 34 |
| Total fat, g/day | 77 ± 18 |
| Saturated fat, g/day | 24 ± 5.6 |
| Added sugar, g/day | 29 ± 19 |
| Total fiber, g/day | 24 ± 12 |
| Sodium, mg/day | 3690 ± 1463 |
| Potassium, mg/day | 2750 ± 996 |
| Calcium, mg/day | 913 ± 268 |
| Magnesium, mg/day | 344 ± 122 |
| Vitamin A (RAE), mcg/day | 646 ± 411 |
| Vitamin C, mg/day | 87 ± 98 |
| Alpha-tocopherol, mg/day | 10.5 ± 4.9 |
| Iron, mg/day | 16 ± 5.9 |
| Zinc, mg/day | 11 ± 3.4 |
| Tryptophan, g/day | 1.2 ± 0.4 |
| Alcohol, g/day | 8.6 ± 22.6 |

Data are expressed as means ± SD. n=27
