# Supplementary material for: Melatonin supplementation does not alter vascular function or oxidative stress in healthy normotensive adults on a high sodium diet
Source: Physiol Rep. 2023 Dec 18;11(24):e15896. doi: 10.14814/phy2.15896 (PMC10727961; doi:10.14814/phy2.15896)
Supplement: Supplementary file 2 — Table S2 [file PHY2-11-e15896-s002.docx]

**Supplemental Table 2**. Average dietary intake of the last 3 days of each intervention

|  | **HSD+PL** | **HSD+MEL** | **P value** |
| --- | --- | --- | --- |
| Energy intake, kcal/day | 2037 ± 715 | 1915 ± 530 | 0.31 |
| Total carbohydrate, g/day | 230 ± 120 | 225 ± 72 | 0.77 |
| Total protein, g/day | 92 ± 46 | 90 ± 51 | 0.74 |
| Total fat, g/day | 81 ± 28 | 74 ± 23 | 0.24 |
| Saturated fat, g/day | 25 ± 9.4 | 23 ± 8.1 | 0.27 |
| Added sugar, g/day | 32 ± 24 | 33 ± 26 | 0.86 |
| Total fiber, g/day | 22 ± 15 | 23 ± 11 | 0.82 |
| Sodium, mg/day | 3346 ± 1456 | 3190 ± 1298 | 0.62 |
| Potassium, mg/day | 2598 ± 1441 | 2533 ± 830 | 0.74 |
| Calcium, mg/day | 909 ± 362 | 865 ± 345 | 0.50 |
| Magnesium, mg/day | 322 ± 151 | 324.9 ± 124 | 0.88 |
| Vit A (RAE), mcg/day | 638 ± 269 | 715 ± 626 | 0.54 |
| Vit C, mg/day | 87 ± 98 | 94 ± 88 | 0.62 |
| Iron, mg/day | 15 ± 6.1 | 14 ± 4.3 | 0.55 |
| Zinc, mg/day | 11 ± 4.8 | 11 ± 3.2 | 0.98 |
| Tryptophan, g/day | 1.1 ± 0.5 | 1.0 ± 0.6 | 0.48 |
| Alcohol, g/day | 3.8 ± 9.7 | 2.5 ± 7.2 | 0.51 |

Data are expressed as means ± SD. HSD+MEL: high sodium diet plus melatonin;

HSD+PL: high sodium diet plus placebo (lactose); Vit, vitamin. n=27
